# A Corticotectal Pathway Regulates Vibrissal Somatosensory-Mediated Predatory Hunting Learning

**DOI:** 10.34133/research.1295

**Published:** 2026-05-25

**Authors:** Yaning Li, Guoqing Chen, Dandan Geng, Rong Zheng, Zhiyong Xie, Tianyun Zhang, Peng Cao, Fan Zhang

**Affiliations:** ^1^ The Key Laboratory of Neural and Vascular Biology, Ministry of Education; The Key Laboratory of Vascular Biology of Hebei Province; Department of Neurobiology, Hebei Medical University, Shijiazhuang, China.; ^2^ National Institute of Biological Sciences; Tsinghua Institute of Multidisciplinary Biomedical Research, Tsinghua University, Beijing, China.; ^3^ Department of Psychological Medicine, Zhongshan Hospital, Institute for Translational Brain Research, State Key Laboratory of Medical Neurobiology, MOE Frontiers Center for Brain Science, MOE Innovative Center for New Drug Development of Immune Inflammatory Diseases, Fudan University, Shanghai, China.

## Abstract

Although predatory behaviors are evolutionarily conserved, they can be enhanced through experience-dependent plasticity. While vibrissal somatosensation is recognized as a key mediator of hunting behavior, the mechanism underlying vibrissal somatosensory-mediated predatory hunting learning remains not yet well understood. In this study, we report that vibrissal tactile input plays an essential role in hunting learning. Activation of the vGlut1^+^ S1BF–SC pathway is sufficient to evoke predatory hunting, while its inactivation abolished practice-induced behavioral improvements. SC-projecting vGlut1^+^ S1BF neurons exhibited robust responses to whisker mechanosensory stimulation. Furthermore, hunting training selectively enhanced α-amino-3-hydroxy-5-methyl-4-isoxazolepropionic acid receptormediated excitatory transmission in the vGlut1^+^ S1BF–SC pathway. Together, these data reveal the corticotectal neural circuit from the primary somatosensory cortex to superior colliculus that was specifically engaged in vibrissal somatosensory-mediated hunting learning by strengthening synaptic connections.

## Introduction

Predatory hunting is evolutionarily conserved across many species as a critical survival strategy, reflecting the enduring principle of natural selection [1]. In rodents, vibrissal tactile inputs constitute a key modality guiding predation behavior, guiding object localization and prey capture [2,3]. Their vibrissae provide essential tactile input that compensates for limited visual information, enabling efficient hunting even in complete darkness [4–6]. Beyond innate instincts, predatory hunting learning in mice markedly enhances both hunting efficiency and behavioral flexibility [7–9]. Through experience, mice refine their sensorimotor coordination and decision-making, enabling adaptive responses to prey variability [10]. However, the mechanism underlying cortical vibrissal somatosensory-mediated predatory hunting learning remains not yet well understood.

The primary somatosensory cortex (S1) dynamically processes tactile information in an experience-, context-, and task-dependent manner [11]. Within S1, the barrel field (S1BF) serves as a specialized hub for processing vibrissal sensory input in rodents. Here, each individual whisker corresponds to a specific cortical barrel unit, allowing precise spatial mapping and functional decoding of tactile information [12,13]. Whisker-derived somatosensory input can be functionally reconstituted at the neural circuit level [14–16]. The primary somatosensory barrel field (S1BF) demonstrates robust experience-dependent plasticity that contributes to tactile perceptual refinement [15,17–19]. Rhythmic whisker stimulation can induce persistent modifications in somatosensory-evoked potentials within the contralateral barrel cortex [20,21]. At the cellular level, synaptic plasticity serves as a fundamental mechanism for vibrissal somatosensory learning, with timing-dependent long-term potentiation (LTP) and long-term depression (LTD) observed in layer II/III pyramidal neurons of rodent S1BF [22,23].

S1BF sends long-range glutamatergic projections to various brain regions, including the superior colliculus (SC) [24,25]. Projections from Cck^+^ Sp5I neurons to the SC constitute a critical neural circuit mechanism for vibrissal somatosensory-evoked predatory hunting [3]. The SC integrates motion-related vibrissal somatosensory and visual signals to generate the motivational drive underlying predatory hunting [24]. Moreover, compared with animals with intact whiskers, mice hunting without whiskers exhibited markedly reduced Fos expression in the lateral SC [26]. A previous study has shown that ocular dominance plasticity in SC was dependent on N-methyl-D-aspartate receptors, the blocking of which partially prevented the deficits of hunting efficiency [27]. However, whether experience-dependent synaptic modifications in the S1BF–SC pathway contribute to vibrissal somatosensory-mediated predatory hunting learning remains unknown.

Our findings demonstrate that vibrissal somatosensory input is critical for the acquisition of hunting behavior. We found that optogenetic activation of the vGlut1^+^ S1BF–SC pathway enhances hunting efficiency, whereas suppression of SC-projecting S1BF neurons impairs somatosensory-mediated predatory hunting learning. This process involves AMPA receptor-dependent synaptic strengthening in trained mice, suggesting that the vGlut1^+^ S1BF–SC corticotectal circuit mediates hunting learning through experience-dependent synaptic plasticity. Beyond their biological importance, such circuit mechanisms of experience-dependent synaptic plasticity may also inform emerging bio-inspired computing paradigms. In recent years, artificial synaptic devices based on memristors and optoelectronic materials have been developed to emulate learning processes observed in biological neural systems [28,29]. These neuromorphic devices can reproduce activity-dependent synaptic modulation, sensory integration, and adaptive computation in hardware platforms [30,31]. Understanding how natural neural circuits integrate sensory signals with hunting learning-dependent synaptic modification therefore provides valuable principles for developing autonomous systems, advancing artificial intelligence, and designing more efficient neuromorphic hardware architectures.

## Results

### Silencing of S1BF impairs vibrissal somatosensory-mediated predatory hunting learning

**Fig. 1.**
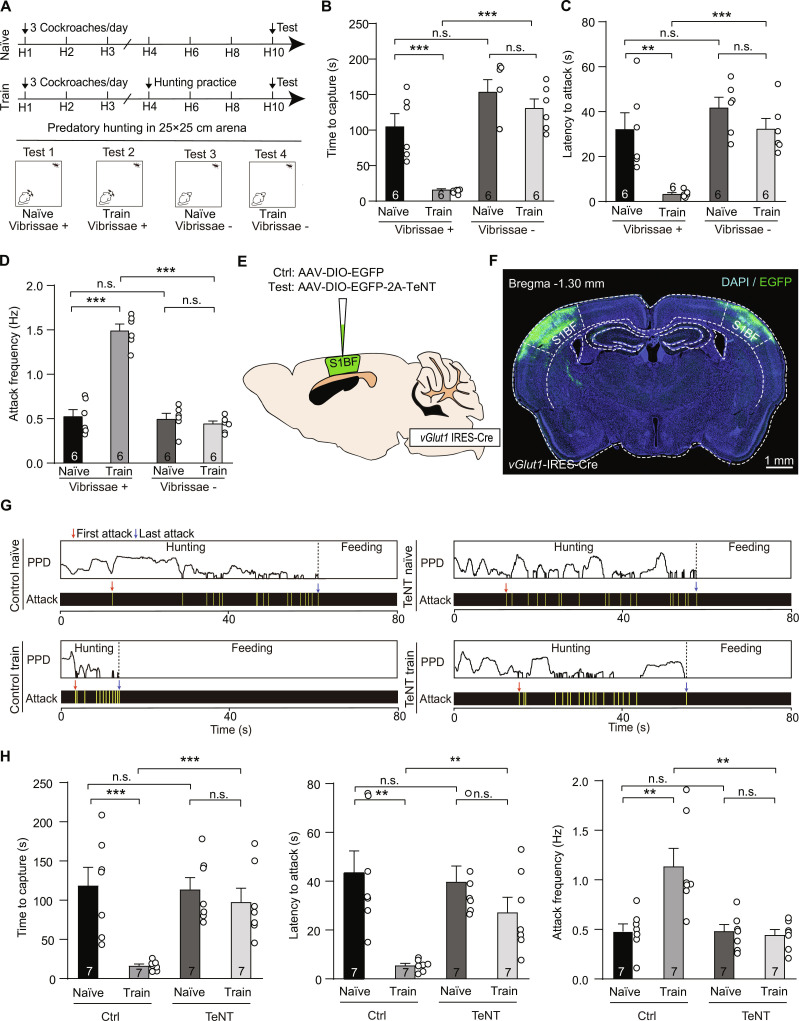
Silencing of the S1BF impairs vibrissal somatosensory-mediated predatory hunting learning. (A) Diagram illustrating the behavioral paradigm used to study predatory hunting learning in mice. (B to D) Quantitative analyses of hunting efficiency (latency to attack, time to capture, and attack frequency) in whisker-intact and whisker-deprived mice. (E) Schematic of the virus strategy used to silence S1BF excitatory neurons. (F) An example coronal section showing TeNT expression in S1BF. (G) Example behavior ethogram of mice with S1BF silence (TeNT) and controls (Ctrl). (H) Quantitative analyses of hunting efficiency (latency to attack, time to capture, and attack frequency) of mice with S1BF silence (TeNT) and controls (Ctrl). Sample sizes (number of mice) are reported in the corresponding graphs. Statistical comparisons were conducted using 2-tailed Student *t* tests (*P < 0.05, **P < 0.01, ***P < 0.001, n.s. > 0.05). Data are shown as mean ± SEM (error bars).

Using a vibrissal somatosensory-triggered predatory hunting paradigm, we examined S1BF’s role in hunting learning by comparing trained and untrained groups (Fig. 1A) [3]. For hunting training, mice completed 3 cockroach-hunting practice trials after days H4, H6, and H8. We tested their predatory hunting performance on day H10. The data in Fig. 1B to D show that hunting practice with vibrissae markedly increased hunting efficiency, as indicated by shorter capture time, reduced attack latency, and higher attack frequency. However, hunting practice without vibrissae had no effect on hunting efficiency. To avoid potential confounding effects of vibrissae trimming on motor function or motivation, we additionally performed reversible local anesthesia of the whisker pad using lidocaine to specifically block vibrissal tactile input. Our results showed that hunting practice also did not affect hunting efficiency (Fig. S1). These results suggest that vibrissal tactile input participates in hunting learning.

Glutamatergic neurons expressing type 1 vesicular glutamate transporter (vGlut1^+^) constitute the largest neuronal population in the S1BF [32]. To silence the output of vGlut1^+^ S1BF neurons, we expressed tetanus neurotoxin (TeNT) (Fig. 1E and F), which specifically blocks neurotransmitter release via proteolytic cleavage of synaptobrevin-2 (Syb2). Synaptic silencing of vGlut1^+^ S1BF neurons abolished hunting practice-induced efficiency improvements, manifesting as increased capture time, prolonged hunting latency, and reduced attack frequency (Fig. 1G and H). These data concluded that S1BF was involved in the regulation of vibrissal somatosensory-mediated predatory hunting learning.

### vGLUT1^+^ S1BF–SC pathway is required for vibrissal somatosensory-mediated predatory hunting learning

**Fig. 2.**
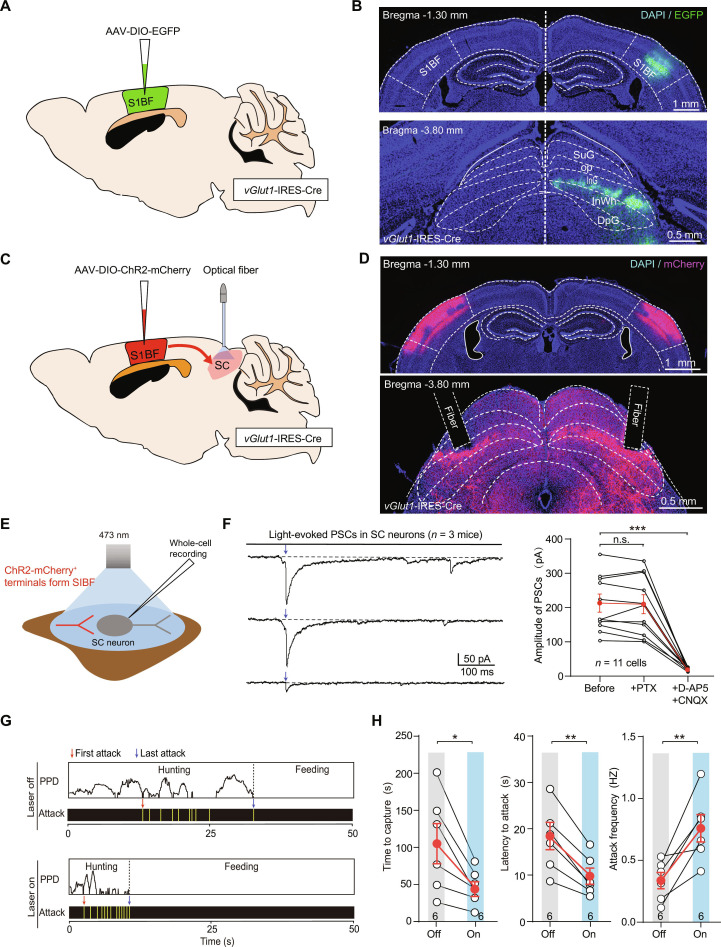
Optogenetic activation of corticotectal pathway promoted hunting efficiency. (A) Schematic of viral injection of AAV-DIO-EGFP into S1BF used to monosynaptic anterograde tracing of S1BF neurons. (B) Micrographs illustrating the expression of EGFP^+^ neurons in S1BF (top) and magnified area of S1BF projections to SC (bottom). (C) Schematic showing bilateral injection of AAV-DIO-ChR2-mCherry into the S1BF and implantation of optical fibers above the intermediate layers of SC to enable cell-type-specific manipulation of the S1BF–SC pathway in *vGlut1*-IRES-Cre mice. (D) Representative coronal sections showing ChR2-mCherry expression in the S1BF (top) and the optical fiber tract positioned above ChR2-mCherry^+^ axon terminals in the intermediate/deep layers of the SC (bottom). (E) Schematic diagram showing the whole-cell recording of light-evoked postsynaptic currents from neurons in the SC. (F) Representative traces and quantitative analyses showing the effects of antagonists of GABAa receptors (picrotoxin [PTX]) and glutamate receptors (D-2-amino-5-phosphonopentanoate [D-AP5]/cyan-quixaline [CNQX]) on the amplitude of light-evoked postsynaptic currents (PSCs) recorded from neurons in the SC. (G) Example behavior ethogram of mice during optogenetic activation of S1BF–SC pathway. (H) Quantitative analyses of hunting efficiency (latency to attack, time to capture, and attack frequency) of mice during optogenetic activation of the vGlut1^+^ S1BF–SC pathway. Statistical analyses were conducted using 2-tailed Student *t* tests (*P < 0.05, *P < 0.01, n.s. > 0.05). Data are shown as mean ± SEM (error bars).

Next, we investigated the output pathways of vGlut1^+^ S1BF neurons by delivering AAV-DIO-EGFP into the S1BF of *vGlut1*-IRES-Cre mice (Fig. 2A). We found that vGlut1^+^ S1BF neurons projected to the SC, which has been previously reported to regulate predatory behavior (Fig. 2B). To evaluate the contribution of the vGlut1^+^ S1BF–SC pathway to vibrissal somatosensory-mediated hunting learning, we injected AAV-DIO-ChR2-mCherry into the S1BF of *vGlut1*-IRES-Cre mice and subsequently implanted optical fibers bilaterally above the SC (Fig. 2C and D). Whole-cell recordings were performed in acute brain slices of the SC (Fig. 2D). Light pulses (473 nm, 2 ms, 20 mW) illuminating ChR2-mCherry axon terminals evoked robust postsynaptic currents (PSCs) from vGlut1^+^ S1BF neurons (213.1 ± 26.6 pA, *n* = 11 neurons), which were inhibited by the perfusion of glutamate receptor antagonists D-2-amino-5-phosphonopentanoate (D-AP5) and cyan-quixaline (CNQX) (Fig. 2E and F). Our results showed that photostimulation of ChR2-mCherry^+^ terminals in the SC robustly facilitated the efficiency of predatory hunting (Fig. 2G and H).

**Fig. 3.**
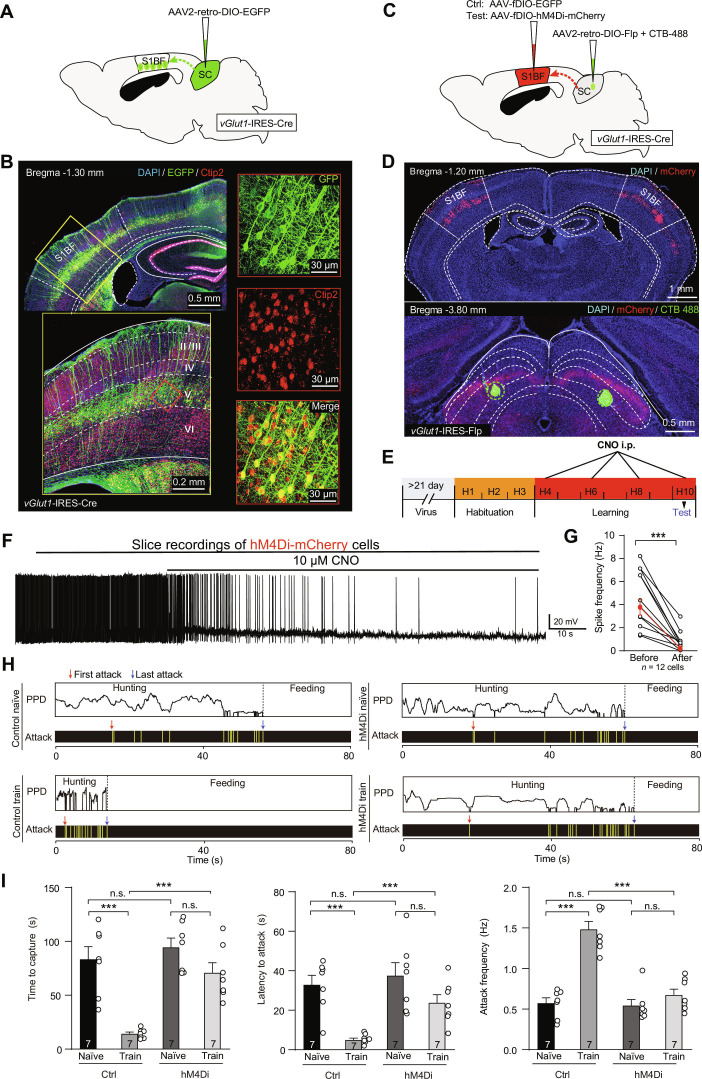
Inhibition of corticotectal pathway abrogated vibrissal somatosensory-mediated predatory hunting learning. (A) Schematic of the antidromic virus strategy used to map the neurons in specific layer V of S1BF projecting to SC in *vGlut1*-IRES-Cre mice. (B) Representative micographs showing the SC projecting S1BF were dually labeled by Ctip2 and EGFP. (C) Diagram illustrating the viral approach employed to specifically inactivate the SC-projecting S1BF neurons in *vGlut1*-IRES-Cre mice. (D) Micrograph of a coronal segment illustrating the expression of hM4Di-mCherry in S1BF (top) and CTB 488 (bottom) in SC. (E) Schematic of the behavioral paradigm of predatory hunting with inactivation of the S1BF–SC pathway in hunting training process (H4 to H10). (F and G) Representative trace of action potential firing (F) and quantitative analysis of the firing rate (G) showing the effectiveness of CNO for chemogenetically silencing hM4Di-expressing S1BF neurons in acute brain slices. (H) Example behavior ethogram of mice with inactivation of the S1BF–SC pathway in hunting D1 to D4. (I) Quantitative analyses of hunting efficiency (latency to attack, time to capture, and attack frequency) in mice with S1BF–SC pathway inactivation during hunting training. Statistical analyses were conducted using 2-tailed Student *t* tests (*P < 0.05, ***P < 0.001, n.s. > 0.05). Data are shown as mean ± SEM (error bars).

To examine connectivity of vGlut1^+^ S1BF neurons, we injected AAV2/2Retro-DIO-EGFP into the SC (Fig. 3A and B). The S1BF is known to be subdivided into 6 anatomically distinct layers (II to VI) based on differences in cell density [33]. Using Ctip2 as a layer V-specific marker [34], we confirmed that retrogradely labeled neurons from the SC were predominantly localized to layer V of S1BF. Through a dual-AAV strategy, AAV2/2Retro-DIO-Flp and CTB-488 were co-injected into the SC while AAV-fDIO-hM4Di-mCherry was injected into the S1BF of *vGlut1*-IRES-Cre mice (Fig. 3C). CTB-488 served as a fiducial marker to verify the precise localization of viral injections in S1BF (Fig. 3D). The effectiveness of CNO in suppressing the firing of action potentials in vGlut1^+^ S1BF neurons was confirmed by slice physiological recordings (Fig. 3F and G). Inactivation of the vGlut1^+^ S1BF–SC pathway markedly impaired hunting practice-induced improvements in predatory efficiency (Fig. 3H and I). Next, we injected AAV2-retro-DIO-Flp into the SC and AAV-fDIO-EGFP-2A-TeNT into the S1BF of *vGlut1*-IRES-Cre mice to silence the terminal fields of the S1BF–SC pathway. We found that silence of the vGlut1^+^ axon terminals in the SC also effectively impaired hunting practice-induced improvements in predatory efficiency (Fig. S2). Collectively, these findings indicate that the vGlut1^+^ S1BF–SC pathway is essential for vibrissal somatosensory-mediated predatory hunting learning.

### SC-projecting vGlut1^+^ S1BF neurons respond to mechanical stimuli

**Fig. 4.**
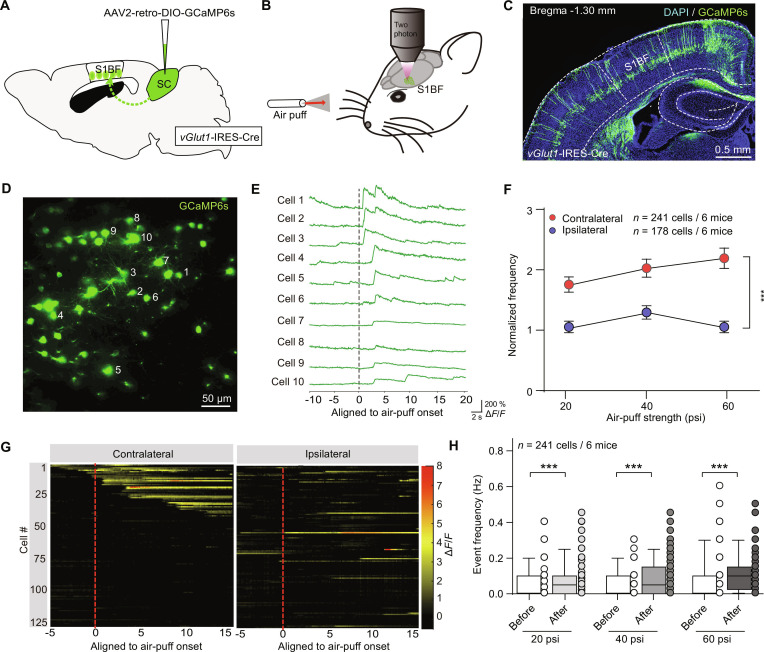
Cortical activation depended on the whisker mechanosensory stimulation. (A) Schematic of antidromic label strategy for 2-photon imaging in S1BF. (B) Example behavior paradigm of 2-photon imaging of S1BF responding to somatosensory signals (air puffs). (C) An example coronal section of GCaMP6s expression after injection of retrograde-labeled AAV into the SC. (D) An example imaging plane consisting of GCaMP6-positive S1BF neurons retrogradely labeled from SC. (E) Example traces of S1BF neurons (white number in D) responding to air-puff stimuli synchronously. (F) Quantitative analyses of normalized somatosensory event frequency to air puffs directing toward contralateral and ipsilateral whiskers and with different strengths. Cell counts: 241 (contralateral) vs. 178 (ipsilateral) from 6 mice. (G) Heatmaps of fluorescence intensity of event activities from corresponding S1BF neurons responding to contralateral (left) and ipsilateral (right) air-puff stimuli. (H) Quantitative analyses of fluorescence event frequency of SC-projecting S1BF neurons before and after contralateral air puff stimuli with different strengths. A total of 241 cells were quantified from 6 mice. Statistical analyses were conducted using 2-tailed Student *t* tests (H) and 2-way ANOVA (F). *P < 0.05, ***P < 0.001. In (H), data are presented using a box-and-whisker plot: the center line denotes the median, the box edges mark the 25th and 75th percentiles, the whiskers extend to the minimum and maximum values, and each point represents an individual measurement. Other data are shown as mean ± SEM (error bars).

Subsequently, we methodically delineated the physiological characteristics of SC-projecting vGlut1^+^ S1BF neurons. To assess their activity during hunting behavior, we conducted 2-photon calcium imaging of GCaMP6s signals in these neurons. Using a retrograde targeting strategy, we injected AAV2/2Retro-DIO-GCaMP6s into the SC and implanted an optical fiber above S1BF in *vGlut1*-IRES-Cre mice (Fig. 4A and B). This approach achieved specific expression of GCaMP6s in SC-projecting vGlut1^+^ S1BF neurons (Fig. 4C).

To mimic whisker sweeping during prey pursuit, we delivered brief air puffs (generated by a Picospritzer) to body-fixed awake mice [3,35]. As expected, a great deal of neurons labeled with GCaMP6 in an imaging window were in an active state in response to air puffs (Fig. 4D and E). Some neurons were synchronized with air-puff stimuli in millisecond-level precision while others were on a delayed time scale (Fig. 4E). Then, we quantified somatosensory responses to graded air-puff intensities (20, 40, and 60 pounds per square inch [psi]) comparing ipsilateral and contralateral sides. GCaMP fluorescence in vGlut1^+^ S1BF neurons exhibited intensity-dependent enhancement, showing markedly greater amplitude in contralateral versus ipsilateral responses (Fig. 4F and G). As quantified, a considerable sharp rise of average fluorescence event frequency after air puffs compared to baseline was visualized in all 241 neurons imaged in contralateral side (Fig. 4H). Taken together, these data proposed a corticotectal encoding mechanism to prey-derived whisker-associated somatosensory cues and a potential neural circuit mediating sensory-triggered hunting behavior.

### Predatory hunting training potentiates AMPAR-mediated synaptic transmission of the vGlut1^+^ S1BF–SC pathway

**Fig. 5.**
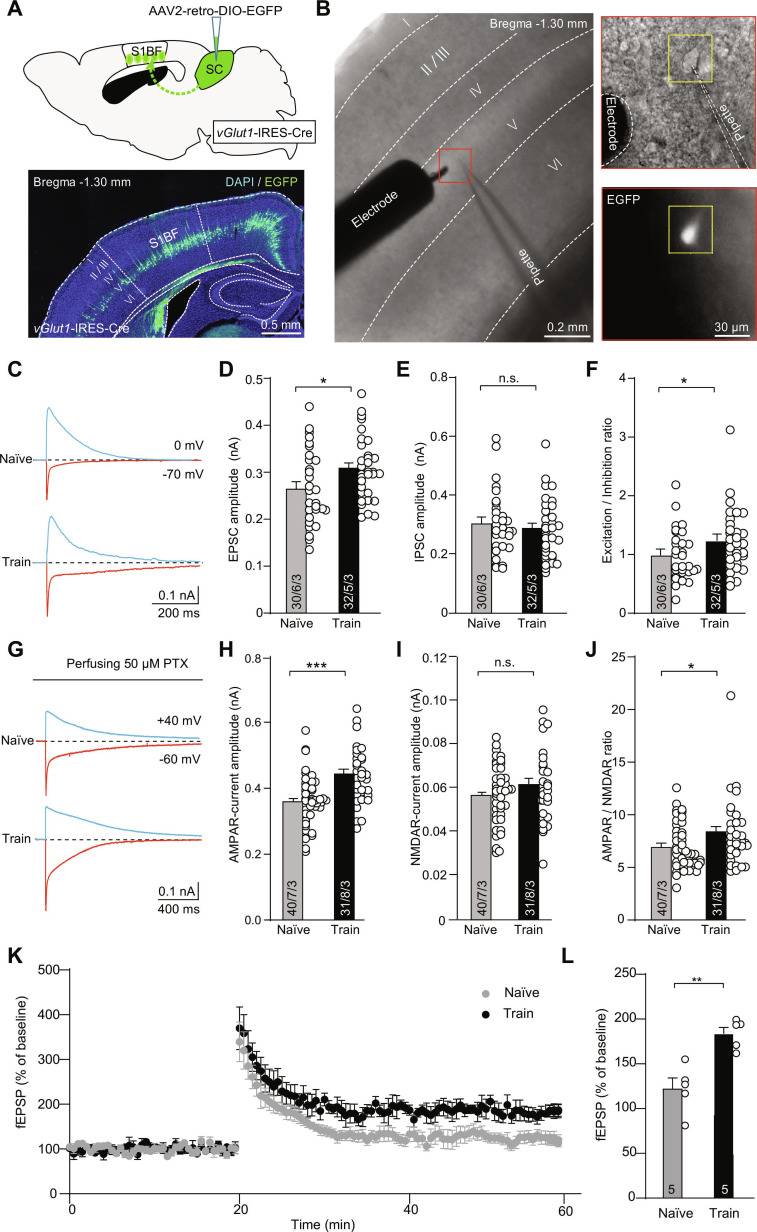
Hunting learning promoted excitatory synaptic transmission of SC-projecting S1BF neurons. (A) Schematic of the strategy used to antidromically label SC-projecting S1BF neurons (top) and example coronal section showing the retrogradely labeled SC-projecting S1BF (bottom). (B) Example micrographs show the location of stimulating electrode and recording pipette during recording under a 4× objective lens (left), and patched neuron expressing EGFP fluorescence under a 20× lens (right). (C) Example traces of EPSCs and IPSCs recorded from naïve and well-trained mice. (D to F) Quantitative analyses of EPSCs, IPSCs, and E/I ratio in naïve and well-trained mice. (G) Example trace of AMPAR-mediated currents and NMDAR-mediated currents recorded from naïve and well-trained mice. (H to J) Quantitative analyses of AMPAR-mediated currents, NMDAR-mediated currents, and AMPAR/NMDAR ratio in naïve and well-trained mice. Numbers of neurons, slices, and mice were indicated from bottom to top in graphs in sequence. (K) Time course of the fEPSP slope in S1BF showing enhanced LTP in the well-trained mice. (L) Quantification of LTP (30 to 40 min), with higher potentiation in the well-trained mice. Statistical analyses were conducted using 2-tailed Student *t* tests (*P < 0.05, **P < 0.01, ***P < 0.001, n.s. > 0.05). Data are shown as mean ± SEM (error bars).

To investigate synaptic plasticity in SC-projecting S1BF neurons following predatory hunting training, we recorded postsynaptic currents from retrogradely labeled layer V neurons. These neurons were identified by injecting AAV2/2Retro-DIO-EGFP into the SC of *vGlut1*-IRES-Cre mice (Fig. 5A and B). Hunting-trained mice exhibited markedly larger excitatory postsynaptic current (EPSC) amplitudes (100 μA stimulation) compared to naïve controls (Fig. 5C and D), while inhibitory postsynaptic currents (IPSCs; Fig. 5E) remained unchanged. The excitation/inhibition (E/I) ratio (Fig. 5F) also experienced an increase. These results suggest experience-dependent potentiation of postsynaptic excitatory transmission. To investigate the molecular mechanisms underlying EPSC differences, we separately analyzed AMPAR- and NMDAR-mediated components of postsynaptic currents. In hunting-trained mice with GABAergic transmission blocked by picrotoxin (PTX; 50 μM), we observed a robust enhancement of AMPAR-mediated EPSCs (Fig. 5G and H), while NMDAR-mediated EPSCs remained unchanged (Fig. 5I). The AMPAR/NMDAR ratio (Fig. 5J) was also elevated, indicating enhanced synaptic connections. Consistently, LTP was markedly enhanced in hunting-trained mice, as indicated by a greater and sustained potentiation of fEPSPs following theta-burst stimulation (TBS) (Fig. 5K and L).

**Fig. 6.**
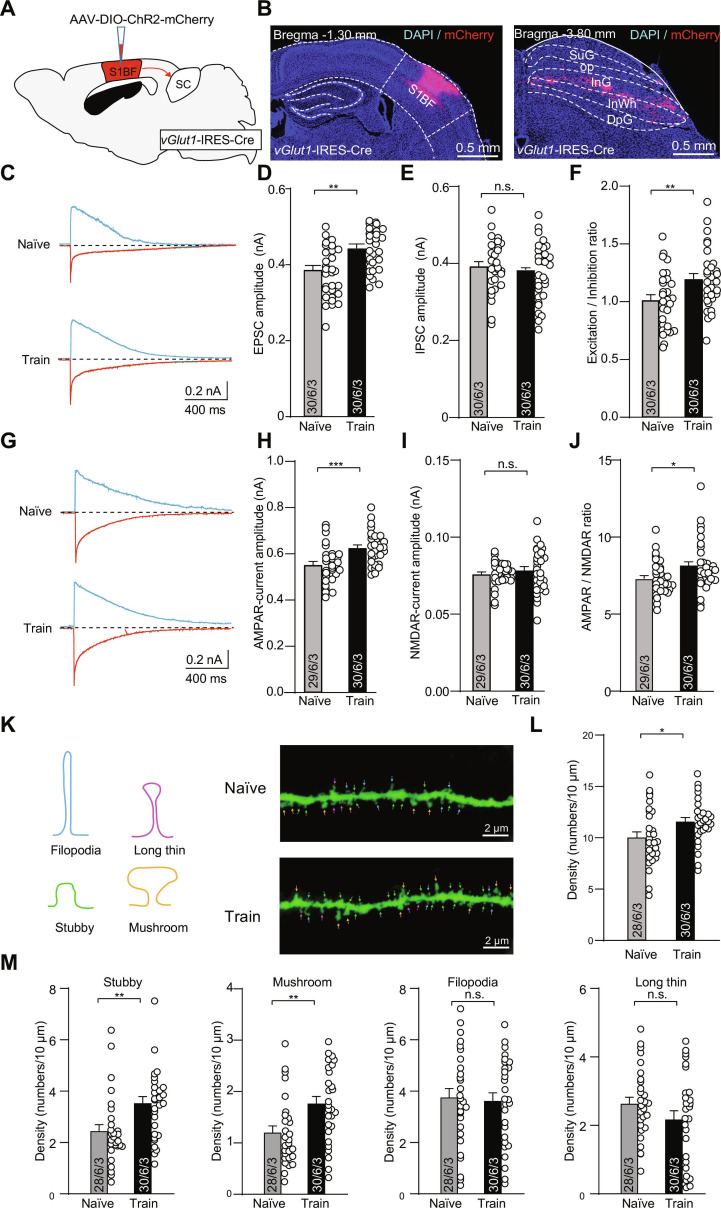
Hunting learning promoted excitatory synaptic transmission in the vGlut1^+^ S1BF–SC pathway. (A) Schematic diagram illustrating the strategy ChR2 injection. (B) Representative coronal sections showing ChR2-mCherry expression in the S1BF (left) and the optical fiber tract positioned above ChR2-mCherry^+^ axon terminals in the intermediate/deep layers of the SC (right). (C) Example traces of EPSCs and IPSCs recorded from naïve and well-trained mice. (D to F) Quantitative analyses of EPSCs, IPSCs, and E/I ratio in naïve and well-trained mice. (G) Example trace of AMPAR-mediated currents and NMDAR-mediated currents recorded from naïve and well-trained mice. (H to J) Quantitative analyses of AMPAR-mediated currents, NMDAR-mediated currents, and AMPAR/NMDAR ratio in naïve and well-trained mice. Numbers of neurons, slices, and mice were indicated from bottom to top in graphs in sequence. (K) Representative confocal stack images showing dendritic spine density in the SC of naïve and well-trained mice. (L) Summary of dendritic spine density in the SC of naïve and well-trained mice. (M) Summary of the density of stubby-, mushroom-, long/thin-, and filopodia-shaped spines in the SC of mice from the above 2 groups. Statistical analyses were conducted using 2-tailed Student *t* tests (*P < 0.05, **P < 0.01, ***P < 0.001, n.s. > 0.05). Data are shown as mean ± SEM (error bars).

To investigate how predatory training affects AMPA receptor dynamics in the SC, we selectively expressed ChR2 in S1BF excitatory neurons of *vGlut1*-IRES-Cre mice by injecting AAV-DIO-ChR2-mCherry and optogenetically stimulated their axon terminals in the SC (Fig. 6A and B). We then performed whole-cell recordings from SC neurons in both naïve and hunting trained animals. We found that hunting training markedly increased the amplitude of optogenetically evoked EPSCs in SC neurons, whereas IPSCs remained unchanged, resulting in an elevated E/I ratio (Fig. 6C to F). Furthermore, analysis of AMPAR- and NMDAR-mediated currents revealed a selective enhancement of AMPAR-mediated currents, while NMDAR-mediated currents remained unchanged. The AMPAR/NMDAR ratio was also elevated (Fig. 6G to J), indicating enhanced synaptic connections. We also found that there was a marked increase in dendritic spine density in SC neurons after hunting training (Fig. 6K and L). High-resolution analysis enabled the classification of synaptic spines into stubby, mushroom, long thin, and filopodia subtypes. Compared with control mice, mice after hunting training exhibited an increased density of stubby- and mushroom-shaped spines, with no obvious effect on long thin and filopodia-like spines (Fig. 6M). Next, we locally infused the AMPAR antagonist CNQX into the SC via bilateral cannula implantation. CNQX infusion markedly impaired prey capture performance after hunting practice, as evidenced by increased attack latency, prolonged capture time, and reduced attack frequency compared to saline-treated controls (Fig. S3). Our data demonstrate that predatory hunting training selectively strengthens AMPAR-mediated excitatory transmission in the vGlut1^+^ S1BF–SC pathway.

## Discussion

While prior work has established the involvement of vibrissae in modulating predation, the neural circuits underlying vibrissae-dependent sensory inputs to guide predatory hunting learning have not been comprehensively investigated. In our study, we identified a critical role for the vGlut1^+^ S1BF–SC pathway in mediating vibrissal somatosensory-dependent predatory hunting learning. SC-projecting S1BF neurons exhibited robust responses to contralateral whisker mechanical stimulation. Enhanced AMPAR-dependent excitatory synaptic transmission in SC-projecting S1BF neurons underlies predatory hunting learning.

The integration of mechanosensory inputs from the vibrissae enhances sensorimotor coordination and decision-making during predatory pursuit, thereby increasing the efficiency and success rate of hunting behaviors. The S1BF has been demonstrated to be essential for processing vibrissal somatosensory information [15,36]. S1BF also demonstrates robust experience-dependent plasticity that facilitates adaptive refinement of tactile perception [17], prompting us to investigate the neural mechanisms underlying vibrissal somatosensory-mediated predatory hunting learning in this region. We first employed a vibrissae-dependent hunting paradigm that eliminated visual cues [3]. Our data showed that hunting training with intact vibrissae markedly improved efficiency, whereas vibrissae-deprived (Fig. 1) and whisker pad anesthesia (Fig. S1) mice exhibited no improvement. Inactivating vGlut1^+^ S1BF neurons abolished the training-induced efficiency enhancement observed in mice with intact vibrissae. The data suggest that vGlut1^+^ S1BF neurons are involved in vibrissal somatosensory-mediated hunting learning.

The SC serves a vital function in predatory hunting by merging visual motion detection and vibrissal somatosensation [37–40]. We found that vGlut1^+^ S1BF neurons send functional monosynaptic projections to the SC. Moreover, selective activation of this vGlut1^+^ S1BF–SC pathway was sufficient to enhance hunting efficiency. SC-projecting vGlut1^+^ S1BF neurons exhibited robust responses to whisker mechanosensory stimulation. Furthermore, retrograde tracing revealed that the SC receives inputs from layer V of S1BF. Layers II/III/V of S1BF are anatomically interconnected with the whisker motor cortex (wM1), a circuit essential for whisker-related tasks [25,41–43]. Inactivation of the vGlut1^+^ S1BF–SC pathway completely abolished training-induced improvements in hunting efficiency (Fig. 3).

Repeated engagement of the vibrissal system during predation facilitates experience-dependent plasticity within sensorimotor circuits. This plasticity may contribute to refinement and learning of effective hunting strategies. Synaptic regulation in hunting learning was evidenced by increased EPSC amplitude in well-trained mice, while IPSC remained unchanged (Fig. 5). Given that cortical networks are predominantly composed of glutamatergic projection neurons [44,45], the retrogradely labeled neurons in S1BF layer V projecting to SC are glutamatergic, which explains the observed EPSC enhancement rather than IPSC changes. Moreover, hunting training selectively enhanced AMPAR-mediated excitatory transmission in SC-projecting S1BF neurons (Fig. 5) and neurons projecting from S1BF to SC (Fig. 6). Additionally, hunting training induces enhanced LTP in S1BF neurons and a marked increase in dendritic spine density within the SC. Taken together, these results establish the essential role of synaptic plasticity in vGlut1^+^ S1BF–SC projections for vibrissal somatosensory-mediated hunting learning.

The present study is limited by its exclusive focus on vGlut1^+^ S1BF–SC projections in regulating vibrissal somatosensory-mediated hunting learning. However, S1BF neuron also projects long-range axons to spinal trigeminal complex (Sp5I) [25], the secondary whisker somatosensory cortex (wS2), and the whisker motor cortex (wM1) [25,46–48]. Sp5 is critical for vibrissal somatosensory-evoked predatory hunting [3,26]. wS2 integrates multiwhisker information through broader receptive fields to enable complex feature extraction and decision-making [49]. wM1 generates motor 48commands for whisker movement during active touch exploration [50]. Future studies should investigate whether S1BF projections to Sp5, wS2, and wM1 participate in regulating vibrissal somatosensory-mediated hunting learning.

## Materials and Methods

### Animals

Experimental procedures adhered to protocols sanctioned by the Administrative Panel on Laboratory Animal Care at the National Institute of Biological Sciences, Beijing. The *vGlut1*-IRES-Cre mice used in this study were acquired from the Jackson Laboratory (JAX Mice and Services). All animals were maintained in standard animal housing facility with constant temperature (20 to 24 °C), humidity (50% to 60%), 12-h light/12-h dark cycle, and cleaning/sterilizing regularly. All animals had free access to the water and food (specific pathogen free criteria) supplied at any time. The mice used were 2 to 5 months old, which could be housed in groups of 3 to 5 mice per cage. All mice used for experiments were kept in one cage for at least 1 week in advance. The mice experiencing virus injection were kept 3 weeks separately before subsequent experiments.

### AAV vectors

Two AAV serotypes (AAV2/9 and AAV2/2Retro) were utilized. Viral preparations were sourced from Shanghai Taitool Bioscience Inc. Stock titers ranged from 0.8 to 1.5 × 10^13^ viral particles/ml, and the working titer for injection was 5 × 10^12^ viral particles/ml.

### Behavior tests

Three- to five-month-old littermates were used as the experimental subjects. All behavioral assays took place between 9:00 AM and 6:00 PM in a room with low illumination. Animals were habituated in the experiment environment 2 h in advance before formal experiment. Before the next test, the apparatus was sprayed and cleaned with 70% (vol/vol) ethanol to eliminate the odor left by the previous animal. Animal behaviors were recorded by video and analyzed using Noldus EthoVision XT software [3].

### Measurement of predatory hunting

The methodology of predatory hunting experiment was performed as previously described [3,35,51]. Behavioral activity of the mice was recorded with 3 orthogonally arranged cameras, with the top-view camera tracking the real-time positions of the predator and prey [35], while the lateral 2 cameras were used to monitor fine movements, such as jaw attack and capture gestures. Before the formal hunting test, the mice underwent a continuous 3-day adaptation (H1, H2, and H3), and 3 to 5 cockroaches (similar size and complete shape) were placed into the home cage of mice with standard chow at a fixed time (usually 2:00 PM). On H3, after consumption of cockroaches, mice were put into a 25 cm × 25 cm square open field arena that they can freely explore. Subsequently, mice experienced training procedure for another 3 days (H4, H6, and H8) with 1 day apart. The chow was restricted at about 1.5 g weight with water available during 24 h before training or testing, and returned to normal diet after mice went back to their home cages. On training day, mice were placed into an open field arena for 10-min habituation. Trial started with the introduction of one cockroach and ended until the mice captured the cockroach and finished ingesting the cockroach. This was repeated three times. The formal test was repeated as the procedure on training day. After all trials finished, 3 key parameters were quantified to judge the hunting efficiency: (a) the latency to attack was measured by the time from the cockroach introduction to the first jaw attack; (b) the time to capture was counted from the first jaw attack to capture; and (c) attack frequency was defined as the attack number divided by capture time during the hunting process [3,35]. In addition, prey–predator distance (PPD) was tracked from overhead cameras. The PPD oscillated overtime and each time close to zero was identified an attack to prey. The final zero indicated the capture of the prey and switch to feeding phase. Measurements from 3 trials were averaged to obtain a single value. Cockroaches were purchased from taobao (www.taobao.com). All hunting processes from the introduction of the cockroach to the end of the trials were recorded by cameras (25 frames per second; Point Grey Research).

For chemogenetic inactivation of the hunting test, the CNO (2 mg/kg) was administered intraperitoneally 30 min in advance of the training and testing on H4 to H10. Other procedures are carried out as standard protocol.

For photostimulation test, 473 nm light-pulse train (20 ms, 10 Hz, 10 mW) was delivered to activate ChR2-mCherry fiber terminals from S1BF in SC through optical fibers.

### AAV stereotaxic microinjection

Anesthesia was induced in mice by intraperitoneal administration of tribromoethanol (125 to 250 mg/kg). Then, the mouse brains were fixed and leveled in the stereotaxic apparatus. The stereotactic coordinates of S1BF were 2 points bilaterally: −1.0 mm anteroposterior, ±3.5 mm mediolateral, and −1.7 mm dorsoventral in relation to bregma. The stereotactic coordinates of SC were as follows: −3.80 mm anteroposterior, ±1.0 mm mediolateral, and −1.5 mm dorsoventral in relation to bregma. A total of 0.5 μl of virus and CTB 488 was infused per site over 10 min using a glass micropipette coupled to a Nanoliter Injector 201 (World Precision Instruments, USA), with a flow rate of 0.10 μl/min to prevent tissue injury [3]. The pipette was withdrawn at least 20 min after viral injection to ensure adequate diffusion and avoid virus loss due to rapid withdrawal. Three weeks were required to recover from surgical trauma.

### Optical fiber implantation

Thirty minutes after virus injection, ceramic ferrules with optical fibers (for optogenetics: 200 μm in diameter, numerical aperture [NA] of 0.37) were stereotaxically implanted into the intermediate layer of SC using denture cement, as follows: −3.8 mm anteroposterior, ±1.2 mm mediolateral, and −1.3 mm dorsoventral in relation to bregma. After surgery, mice were returned to their home cages for recovery for at least 3 weeks.

### Slice electrophysiological recording

Anesthesia was induced in mice by intraperitoneal injection of 20% isoflurane. Decapitate the animal and scoop out the intact brain into a breaker of pre-chilled and oxygen-saturated cutting buffer (in mM: 228 sucrose, 26 NaHCO_3_, 11 glucose, 2.5 KCl, 7 MgSO_4_, 1 NaH_2_PO_4_, and 0.5 CaCl_2_, pH 7.25, 330 to 340 mOsm), allowing the brain to cool evenly for ~1 min [35]. The brain was trimmed and affixed on the specimen holder using adhesive glue. The reservoir was filled with the above cutting buffer. Mouse brain was sliced (300 μm) in a vibratome with the remaining pre-chilled cutting buffer. Subsequently, the slices were transformed to oxygenated artificial cerebrospinal fluid (ACSF [in mM]: 125 NaCl, 26 NaHCO_3_, 2.5 KCl, 1.3 MgSO_4_, 10 glucose, 1 NaH_2_PO_4_, and 2.5 CaCl_2_, pH 7.25, 300 to 310 mOsm) at 28 °C for 1 h before transferring to the recording chamber at room temperature [35]. The entire procedure was continuously supplied with mixed gas (5% CO_2_ and 95% O_2_) to maintain oxygen saturation. During recording, slices were immersed in a chamber with oxygenated ACSF at a rate of 2 ml/min in room temperature. Acute brain slices were imaged using a 40× Olympus water-immersion objective, differential interference contrast (DIC) optics, and a charge-coupled device camera. Patch pipettes were fabricated from borosilicate glass capillaries using a PC-10 pipette puller [33]. To record the general postsynaptic current of PSCs (voltage clamp), pipettes were filled with a low Cl^−^ internal solution (in mM: 126 CsMeSO_3_, 10 HEPES, 1 EGTA, 1 CaCl_2_, 1 Na-GTP, 4 Mg-ATP, and 2 QX314, pH 7.4, 290 to 300 mOsm). EPSCs were recorded at a holding potential of −70 mV, while IPSCs were recorded at 0 mV. Similarly, to record AMPA and NMDAR-mediated EPSCs, pipettes were filled with another internal solution (in mM: 117.5 CsMeSO_3_, 15.5 CsCl, 10 TEA-Cl, 10 HEPES, 10 EGTA, 8 NaCl, 1 MgCl_2_, 0.3 Na-GTP, 4 Mg-ATP, 1 QX314, and 10 Na phosphocreatine, pH 7.4, 290 to 300 mOsm). AMPA receptor-mediated EPSCs were recorded at a holding potential of −60 mV, while NMDA receptor-mediated EPSCs were recorded at +40 mV. PSCs were evoked by 100-μA stimulus intensity delivering from the stimulating electrode (FHC bipolar platinum), which positioned over patched S1BF neurons retrogradely labeled with EGFP around ~100 μm away. AMPAR- and NMDAR-mediated PSCs were recorded in the presence of PTX (50 μM) for blocking the GABA receptors. Light-evoked PSCs from ChR2-mCherry^+^ SC were triggered by single light pulses (473 nm, 2 ms, 20 mW) in the presence of 4-aminopyridine (20 μM) and tetrodotoxin (1 μM). Antagonists of GABAa receptors (PTX) and glutamate receptors (D-AP5/CNQX) were perfused with ACSF to examine the neurotransmitter receptor type of optically evoked postsynaptic currents. The resistance of pipettes varied between 4.0 and 5.5 MΩ, and the recorded data series resistance value is less than 15 MΩ. Recording data were filtered at 1 to 2 kHz and digitized at 10 kHz via a Digidata 1440A. Data acquisition was performed with Clampex 10, and subsequent analysis was conducted using Clampfit 10. The E/I ratios were determined by dividing the EPSC charge by IPSC charge.

### Immunohistochemistry

Anesthesia was induced in mice by intraperitoneal injection of 20% isoflurane. Brain was removed after perfusing transcardially with 0.9% saline and 4% paraformaldehyde, and sank in phosphate-buffered saline (PBS) containing 30% sucrose. Cryostat sections (40 μm) containing the SC and S1BF were collected and blocked with blocking solution (PBS containing 10% donkey serum and 0.7% Triton X-100) for 2 h [35]. Then these slices were incubated with primary antibodies (rabbit anti-mCherry [1:500, Abcam, UK], rabbit anti-GFP [1:500, Abcam], and rat anti-Ctip2 [1:500, Abcam]) for 6 h at room temperature. Next, the primary antibodies were washed 3 times with washing buffer (1×PBST, PBS containing 10% donkey serum, and 0.7% Triton X-100). Subsequently, they were incubated with Alexa Fluor 550 donkey anti-rabbit IgG (1:1,000, Invitrogen, USA), Alexa Fluor 488 donkey anti-rabbit IgG (1:1,000, Invitrogen), and Alexa Fluor 550 donkey anti-rat IgG for another 2 h. After washing 3 times with 1×PBST, sections were stained with 4,6-diaminodino-2-phenylindole (Invitrogen, 1:20,000) for 15 min [35], following one time PBS washing. Mounted sections on SuperFrost slides were imaged using either an Olympus VS120 epifluorescence system (10× objective) or a Zeiss LSM800 confocal microscope with a 60× oil-immersion objective. Excitation was performed sequentially with 488-, 543-, and 633-nm lasers to prevent channel bleed-through. Pixel saturation was avoided via Hi-Lo monitoring, and image analysis was conducted in ImageJ.

### In vivo 2-photon imaging

Cranial surgery for 2-photon imaging was performed as previously described, with minor modifications [52,53]. Briefly, 3 weeks after AAV2/2Retro-DIO-GCaMP6s injection in SC of *vGlut1*-IRES-Cre mice, the mice were implanted with a cranial window over S1BF (2.5 mm diameter; center relative to bregma: lateral, 3.5 mm; rostral, 1.0 mm). Mice were anesthetized with Avertin (0.25 g/kg, i.p.), and following the excision of the skin and musculature from the skull, a custom-designed chamber was affixed to the skull using cyanoacrylate and strengthened with dental cement for head-fixed 2-photon imaging [53]. Craniotomies were performed to make a 2.5 cm × 2.5 cm square size hole in the skull over the S1BF region with a high-speed drill (RWD, China). The dura was carefully removed with thin forceps avoiding damage to blood vessels and tissues, and a 2.5 cm × 2.5 cm square glass coverslip was placed on the cranial window. Two-photon imaging of the mice was conducted after 3 days’ recovery. Two-photon imaging was conducted using a 2-photon microscope (Nikon A1+MP, Japan) outfitted with a resonant scanning module (Thorlabs, USA), GaAsP photomultiplier tubes (Hamamatsu, Japan), and a 25× 1.1 NA microscope objective (Nikon, Japan). The 920-nm excitation light was produced with a Ti:sapphire laser (Mai Tai DeepSee, Spectra-Physics, USA). Imaging fields were confined to the region exhibiting GCaMP6s expression in S1BF at a depth of 500 μm beneath the pial surface at a frequency of 7.7 Hz.

Air puffs of 50 ms duration and 20, 40, or 60 psi strength were applied through a 1.5-mm-diameter metal tube coupled to a Picospritzer III. Stimulation timing was controlled by a programmable pulse generator. During whisker stimulation, the tube was oriented from the temporal toward the nasal side of the mouse, with a distance of ~20 mm between nozzle and whiskers [35]. For each strength, 3 trials were repeatedly presented to the whiskers. Fluorescence intensity changes (Δ*F*/*F*) in Ca^2+^ transients in layer V of S1BF greater than 1 are defined as one standard deviation (SD) event. Events were recorded 10 s before air puff stimuli following 20 s afterwards. Recordings were examined via bespoke software (MATLAB) and ImageJ.

The event frequency was defined as event numbers divided by durations (s). The normalized frequency was defined as event frequency after air puff stimuli divided by average event frequency before.

### Golgi staining

See the Supplementary Methods for details.

### Electrophysiological recording of LTP

See the Supplementary Methods for details.

### Data analysis

All the results were expressed as mean ± SEM. The graphs were plotted and analyzed by GraphPad Prism 6.0. Data of 2 photons were presented as a box-and-whisker plot, in which the central line signifies the median, the borders of the box represent the 5th and 95th percentiles, the whiskers illustrate the minimum and maximum values, and individual data points are depicted as dots. One-way ANOVA was used for analyses involving more than 2 groups, and Student *t*-tests were applied for 2 groups. The *P* < 0.05 generated by the statistical method was revealed as statistically significant.

## Data Availability

All data presented in this research will be made available by the corresponding authors upon request. Any supplementary information necessary for the reanalysis of the data presented in this paper can be obtained from the corresponding authors upon request. The MATLAB code for data analysis can be obtained from the appropriate author upon request.
