# Supplementary material for: A Corticotectal Pathway Regulates Vibrissal Somatosensory-Mediated Predatory Hunting Learning
Source: Research (Wash D C). 2026 May 25;9:1295. doi: 10.34133/research.1295 (PMC13199649; doi:10.34133/research.1295)
Supplement: Supplementary 1 — Figs. S1 to S3 Movies S1 to S5 Supplemental Methods Table S1 [file research.1295.f1.zip › renamed_fd9b7.docx]

**Supplementary Materials**

**A Corticotectal Pathway Regulates Vibrissal Somatosensory Mediated Predatory Hunting Learning**

Yaning Li^1†^, Guoqing Chen ^2†^, Dandan Geng^2†^, Rong Zheng^1^, Zhiyong Xie^3^, Tianyun Zhang^1^, Peng Cao^2*^, Fan Zhang^1*^

1 The Key Laboratory of Neural and Vascular Biology, Ministry of Education; The Key Laboratory of Vascular Biology of Hebei Province; Department of Neurobiology, Hebei Medical University, Shijiazhuang, China

2 National Institute of Biological Sciences; Tsinghua Institute of Multidisciplinary Biomedical Research, Tsinghua University, Beijing, China

3 Department of Psychological Medicine, Zhongshan Hospital, Institute for Translational Brain Research, State Key Laboratory of Medical Neurobiology, MOE Frontiers Center for Brain Science, MOE Innovative Center for New Drug Development of Immune Inflammatory Diseases, Fudan University, Shanghai, China

†These authors contributed equally to this work.

*These authors jointly supervised this work: Fan Zhang, Peng Cao

*Correspondence to:

Fan Zhang, Ph.D.

Department of Biochemistry and Molecular Biology, Hebei Medical University, Shijiazhuang, China. 050017

zhangfan86@hebmu.edu.cn

Peng Cao, Ph.D

National Institute of Biological Sciences, Beijing, China.102200

caopeng@nibs.ac.cn

This file includes:

Supplementary Figure1 to 3

Supplementary Video 1 to 5

Supplemental Methods

Supplementary Tables Reagents

**
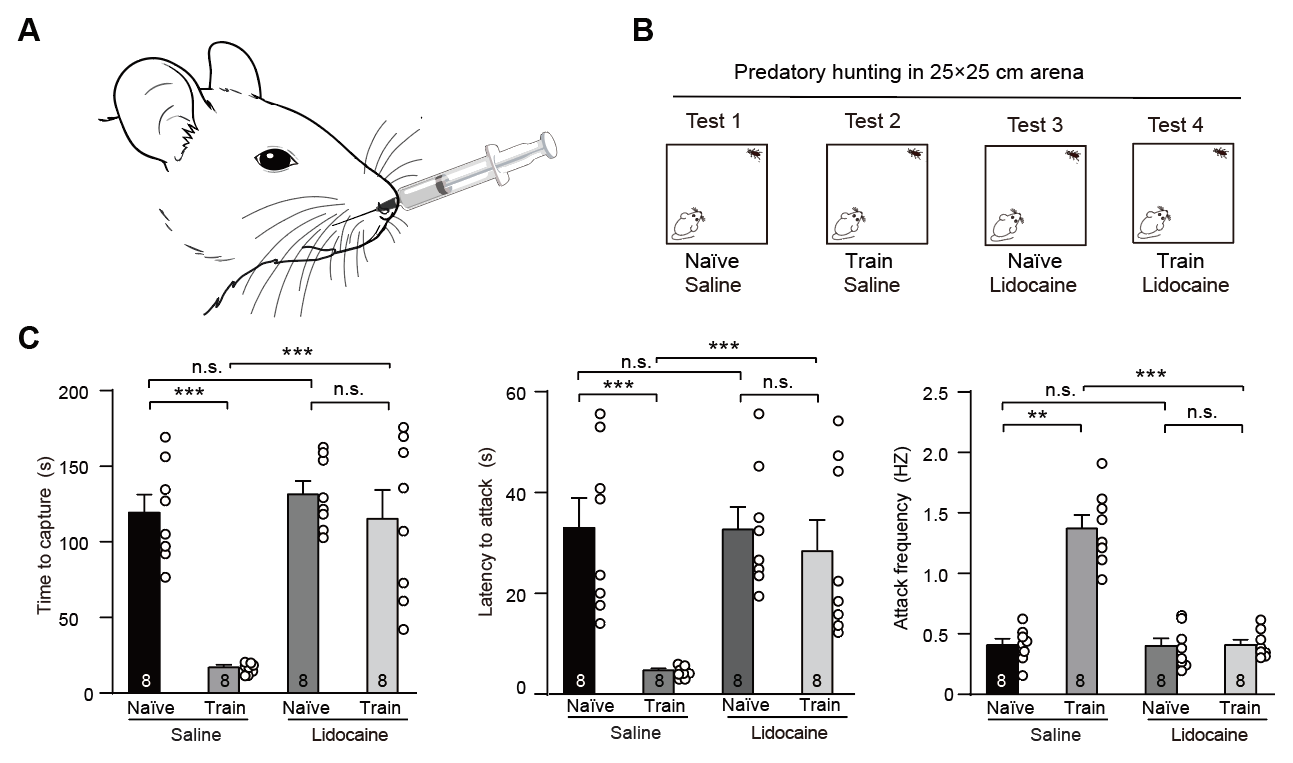
**

**Supplemental Figure 1: Reversible whisker pad anesthesia impairs vibrissal somatosensory mediated predatory hunting learning**

**(A, B)** Schematic diagram of lidocaine injection into the whisker pads of mice. **(C)** Quantitative analyses of hunting efficiency (latency to attack, time to capture and attack frequency) of mice with whisker pad anesthesia (Lidocaine) and controls (Ctrl). Sample sizes (number of mice) are reported in the corresponding graphs. Statistical comparisons were conducted using two-tailed Student’s t-tests (**p* < 0.05, ***p* < 0.01, ****p* < 0.001, *n.s*. > 0.05). Data are shown as mean ± SEM (error bars)

**
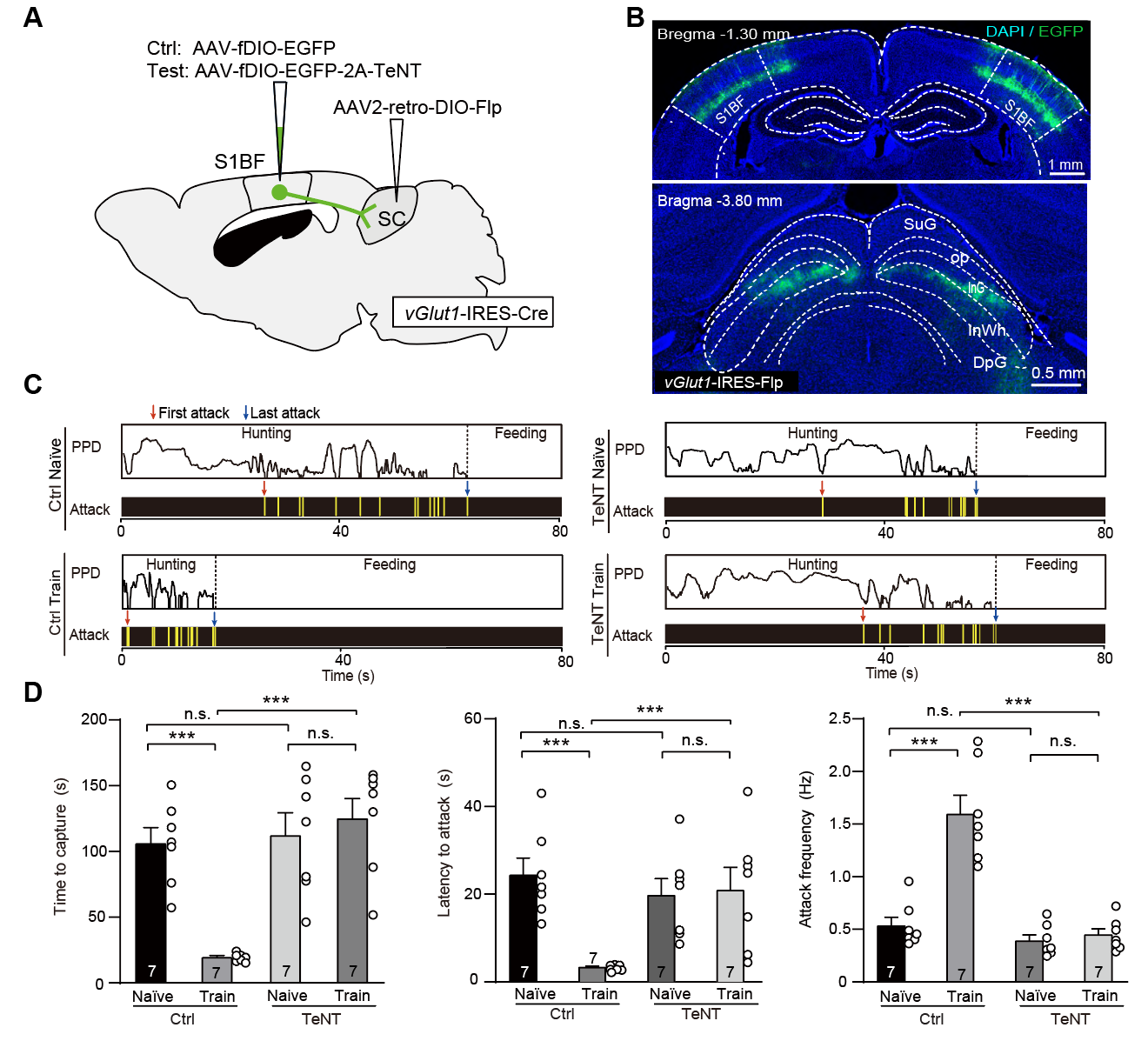
**

**Supplemental Figure 2: Silencing of the terminal fields of the vGlut1⁺ S1BF-SC pathway impairs vibrissal somatosensory mediated predatory hunting learning**

**(A)** Diagram illustrating the viral approach employed to specifically silence the terminal fields of the vGlut1^+^ S1BF-SC pathway in *vGlut1*-IRES-Cre mice. **(B)** Micrograph of a coronal segment illustrating the expression of TeNT in S1BF (*top*) and SC (*bottom*). **(C)** Example behavior ethogram of mice with silencing of S1BF-SC pathway in hunting. **(D)** Quantitative analyses of hunting efficiency (latency to attack, time to capture and attack frequency) in mice with or without TeNT silence of the vGlut1⁺ S1BF-SC pathway. Statistical analyses were conducted using two-tailed Student’s t-tests (**p* < 0.05, ****p* < 0.001, *n.s.* > 0.05). Data are shown as mean ± SEM (error bars).

**
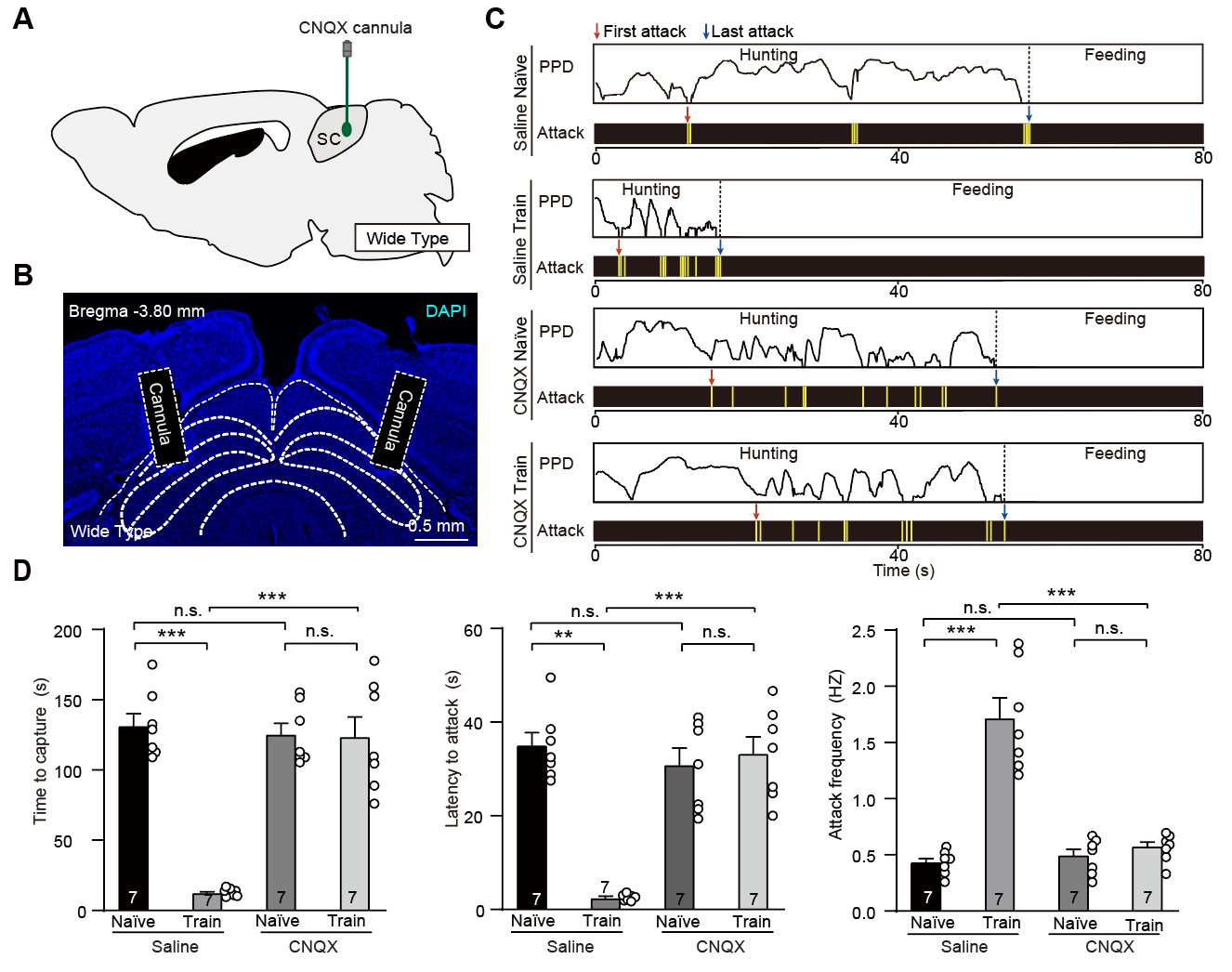
**

**Supplemental Figure 3: AMPAR blockade in SC impairs vibrissal somatosensory mediated predatory hunting learning**

**(A)** Schematic illustration of cannula implantation into SC of wild-type mice for local infusion of CNQX. **(B)** Representative coronal section showing the cannula placement in the SC. **(C)** Representative examples of hunting behavior in mice receiving intra-SC CNQX infusion. **(D)** Quantitative analyses of hunting efficiency (latency to attack, time to capture, and attack frequency) in mice with saline or CNQX infusion into the SC. Statistical analyses were conducted using two-tailed Student’s t-tests (**p* < 0.05, ****p* < 0.001, *n.s.* > 0.05). Data are shown as mean ± SEM (error bars).

**Supplementary Video 1. Hunting learning improved hunting efficiency with intact whiskers in mice**

Analysis of predatory hunting behavior in a representative mouse with intact whiskers before and after hunting learning.

**Supplementary Video 2. Hunting learning had no effect on hunting efficiency without intact whiskers in mice**

Analysis of predatory hunting behavior in a representative mouse with removed whiskers before and after hunting learning.

**Supplementary Video 3. Control mice with TeNT siliensing of vGlut1⁺ S1BF neurons showed enhanced hunting efficiency after hunting learning**

Analysis of predatory hunting behavior in a control mouse with TeNT-mediated S1BF silencing before and after hunting learning.

**Supplementary Video** **4. TeNT-mediated inactivation of vGlut1⁺ S1BF neurons abolished hunting learning induced hunting efficiency enhancement**

Analysis of predatory hunting behavior in a representative mouse with TeNT-mediated S1BF silencing before and after hunting learning.

**Supplementary Video 5. Optogenetic Activation of vGlut1⁺ S1BF–SC pathway promotes hunting**

Behavioral analysis of predatory hunting in a representative mouse with and without photostimulation of the vGlut1⁺ S1BF–SC pathway.

**Supplemental Methods**

**Golgi staining**

To visualize neuronal morphology, mice in the control (untrained) and test (predatory trained) groups were sacrificed, and fresh brain tissues were harvested immediately. The brains were rinsed with distilled water and immersed in Golgi-Cox fixative (containing 5% potassium chromate, 5% potassium dichromate, and 5% mercuric chloride). The samples were incubated at room temperature in the dark for 2 days. Subsequently, the fixative was replaced with fresh Golgi-Cox solution after an initial phosphate-buffered saline (PBS) wash. The impregnation process lasted 14 days in a cool, ventilated, and dark environment, with the solution refreshed after the first 48 hours and every 3 days thereafter. Following impregnation, the brains were rinsed several times with distilled water. Coronal sections (60 μm thick) were obtained using a vibrating microtome (tissue secured with cyanoacrylate adhesive and immersed in distilled water). Sections were mounted onto glass slides using a fine brush, blotted with filter paper, and dried overnight at 4°C. For visualization, the slides were rinsed with ultra-pure water (5 min), developed in concentrated ammonia (10 min), and rinsed twice more. Finally, the sections were coverslipped using glycerine gelatin and stored in the dark at room temperature. Dendritic morphology was characterized using a Zeiss LSM 800 confocal microscope (Carl Zeiss, Germany). Images were taken by z-stack scanning with the excitation wavelength of 405 nm, and then the virtual color was converted into green color. For quantitative assessment of dendritic complexity, Sholl analysis was performed. A series of concentric circles, centered on the soma with a radial increment of 10 μm, were superimposed over the dendritic tree.Consistent with previous studies, further high-resolution analysis enabled classifying synaptic spines into long thin, filopodia, stubby and mushroom subtypes. Dendritic spine classification was then completed in the Imaris spine classification module. In brief, spines with length < 1 μm are categorized as stubby spines, those with length < 3 μm and max head width > mean of neck width*2 are categorized as mushroom spines, spines with mean of head width ≥ mean of neck width are categorized as long thin type, filopodia spines are those except the above three types.

**Electrophysiological recording of LTP**

Following incubation, brain slices were transferred to a recording chamber and continuously perfused with oxygenated ACSF (95% O2/5% CO2) at a flow rate of 2 mL/min. The perfusion temperature was maintained at 30–32°C using a temperature controller. Slices containing the primary somatosensory cortex barrel field (S1BF) were visualized under an upright microscope. Field excitatory postsynaptic potentials (fEPSPs) were recorded using glass microelectrodes (2–5 MΩ) filled with ACSF. To assess synaptic transmission in the S1BF, a bipolar stimulating electrode was placed in layer IV and the recording electrode was positioned in layer V. An input–output (I/O) curve was first generated to determine the maximal fEPSP response, and baseline stimulation intensity was adjusted to evoke approximately 50% of the maximal response. Baseline fEPSPs were recorded for 20 min at 0.033 Hz. Long-term potentiation (LTP) was induced by theta-burst stimulation (TBS), consisting of 10 bursts of 4 pulses at 100 Hz, delivered at 5 Hz, at an intensity eliciting 75% of the maximal fEPSP response. After TBS, fEPSPs were continuously recorded for 60 min at the baseline stimulation intensity. LTP magnitude was quantified as the average fEPSP slope during the final 10 min of recording and normalized to the 20 min pre-TBS baseline.

**Lidocaine-anaesthetised whisker pad**

Following previously described procedures, 1% lidocaine hydrochloride was subcutaneously injected into the bilateral vibrissal pads (15 μL per side). Behavioral experiments were initiated approximately 10 min after injection, during which whisker-evoked tactile responses were effectively suppressed. Control animals received equivalent volumes of saline.

**Cannula implantation and drug infusion**

A guide cannula was stereotaxically implanted above the SC. The cannula had an inner diameter of 150 μm and an outer diameter of 300 μm, and was secured to the skull using acrylic cement. For drug infusion, the cannula was connected to a catheter prefilled with either CNQX (50 nM) or saline. The catheter was attached to a Hamilton syringe mounted on an infusion pump to control delivery. CNQX or saline was infused into the SC at a rate of 50 nL/min, with a total volume of 200 nL per infusion. Vibrissal somatosensory mediated predatory hunting learning was assessed after each infusion.

**Table S1 Information of mouse lines and reagents**

| REAGENT or RESOURSE | SOURCE | IDENTIFIEY |
| --- | --- | --- |
| **Antibodies** | | |
| rabbit anti-GFP | Abcam | Cat # ab290 |
| rabbit anti-mCherry | Abcam | Cat # ab167453 |
| rat anti-Ctip2 | Abcam | Cat # ab18465 |
| Alexa Fluor 550 donkey anti-rabbit | Invitrogen | Cat # A31570 |
| Alexa Fluor 488 donkey anti-rabbit | Invitrogen | Cat # A21206 |
| Alexa Fluor 550 donkey anti-rat | Invitrogen | Cat # A21202 |
| **Bacterial and virus strains** | | |
| AAV2/9-CMV-DIO-EGFP-P2A-TetTox-WPRE-pA | TaiTool Co., Ltd | Packaged by TaiTool Co., Ltd., China |
| AAV2/9-hSyn-EGFP-WPRE-pA | TaiTool Co., Ltd | S0237-9-H5 |
| AAV2/9- Ef1α-DIO-hChR2-mCherry-WPRE-pA | TaiTool Co., Ltd | Cat # S0170-9-H50 |
| scAAV2/2Retro-hSyn-FLEX-Flpo-pA | TaiTool Co., Ltd | Cat # S0293-2R-H20 |
| AAV2/9-Ef1α-fDIO-EGFP-P2A-TetTox-WPRE-pA | TaiTool Co., Ltd | Cat # S0551-9-H20 |
| AAV2/9-hEF1a-fDIO-EGFP-WPRE-pA | TaiTool Co., Ltd | Cat # S0253-9-H50 |
| scAAV2/2Retro-hSyn-EGFP-WPRE-pA​​ | TaiTool Co., Ltd | Cat # S0581-2R-H20 |
| AAV2/2Retro-Plus-hSyn-FLEX-GCaMP6s-WPRE-pA | TaiTool Co., Ltd | S0226-2RP-H20 |
| **Chemicals** | | |
| D-AP5 | Tocris | Cat # 0106 |
| CNQX | Tocris | Cat # 0190 |
| Picrotoxin | Tocris | Cat # 1128 |
| DAPI | Sigma | Cat # D8417 |
| CTB-488 | Thermo Fisher  Scientific | Cat # C34776 |
| CNO | Enzo Life Science | Cat # BML-NS105 |
| 4-AP | Sigma | Cat # 504-24-5 |
| TTX | Sigma | Cat # 4368-28-9 |
